# Traits analysis of an aphid-killing *Pantoea agglomerans* strain for use in biological control

**DOI:** 10.3389/finsc.2026.1896449

**Published:** 2026-08-14

**Authors:** Alisa Hamidović, Nell Ghisdal, Linda Dhondt, Inès Pons, Thomas Dagbert, Mey Jerbi, Jérôme Ambroise, Bertrand Bearzatto, Jean-Luc Gala, Thierry Hance, François Renoz

**Affiliations:** 1 Biodiversity Research Centre, Earth and Life Institute, UCLouvain, Louvain-la-Neuve, Belgium; 2 Mutualisms Research Group, Max Planck Institute for Biology, Tübingen, Germany; 3 Plant Ecology, ecoPhysiology and Sustainable Agriculture (PEPA) Lab: Plant Ecology, ecoPhysiology and Sustainable Agriculture, Earth and Life Institute, UCLouvain, Louvain-la-Neuve, Belgium; 4 Center for Applied Molecular Technologies, Institute of Experimental and Clinical Research, UCLouvain, Woluwe-Saint-Lambert, Belgium

**Keywords:** Aphis fabae, biocontrol, biopesticide, endophyte, entomopathogen, *Pantoea*, phytopathogen, Vicia faba

## Abstract

**Introduction:**

In crop pest control, microbial bioinsecticides are considered promising for overcoming the critical limitations of traditional chemical pesticides regarding environmental and health impact. In this study, we report a strain of *Pantoea* agglomerans (ELIV) lethal to black bean aphid Aphis fabae: we investigated the effects of this bacterium on aphids and host plants to test its potential as a natural insecticide.

**Methods and Results:**

We found that the entomopathogenic properties of *P. agglomerans* ELIV occur only through ingestion via oral infection assays, with no pathogenicity by contact observed via spraying tests. The bacterium does not appear to circulate within the plant after watering, remaining at the root level and having only slight effects on tested plant growth parameters. Genome analysis of *P. agglomerans* ELIV reveals its richness in genes encoding virulence factors, particularly iron-chelating siderophores, possibly explaining its ability to rapidly kill infected insects.

**Discussion:**

Overall, our results present the strengths and limitations of the species *P. agglomerans* for the development of new bioinsecticides. Although the use of *P. agglomerans* may be of limited interest for controlling piercing-sucking insects as they feed internally, it may be relevant against chewing insect pests, e.g. lepidopteran caterpillars and beetles.

## 1 Introduction

Due to its harmful effects on human health and environment, many countries have committed to phasing out synthetic chemical pesticides, for which alternatives can be found in biopesticides (1, 2), *i.e.* a range of natural living substances and metabolic products used to control pest populations (3). Entomopathogenic bacteria are promising alternatives for controlling insect pests (4–8), a notable case being *Bacillus thuringiensis*, which accounts for ~90% of current bacterial biopesticides (9, 10). Although other promising bacterial candidates have been identified *e.g. Pseudomonas fluorescens*, *Serratia marcescens*, *Brevibacillus laterosporus*, and *Bacillus subtilis*, their modes of action remain less understood (11–14). In fact, the bacterial world is teeming with candidates with untapped entomopathogenic potential for dealing with the growing resistance of insect pests to current insecticides (15–17).

The genus *Pantoea* (Enterobacteriaceae) is particularly interesting for identifying new candidates for insect biological control, as it is extremely widespread and includes members with entomopathogenic properties (18). Currently comprising more than 25 recognized species (19), this genus of yellow-pigmented, rodshaped Gram-negative aerobic bacteria displays diverse lifestyles in a wide range of aquatic environments and is often associated with animals (20). *Pantoea agglomerans* is the most commonly isolated species of this genus, and its effects on its hosts and environment are highly diverse and strain-dependent (21–25). Some strains have been identified as opportunistic human pathogens (26), others as plant diseases agents (27–30), or even epi- or endophytic mutualists in plants, providing protection against pathogens or acting as plant growth promoters (31–33) by participating in atmospheric nitrogen fixation, phytohormone production, phytate degradation, phosphate and ammonium solubilization, *etc.* (25). *Pantoea agglomerans* often forms part of the microbiota of many insects, including bees (34, 35), ants (36), termites (37), wood-boring beetles (38), fruit flies (39) and many others (20).

Despite the pervasiveness and versatility of *P. agglomerans*, the nature of this bacterium’s interaction with insects remains difficult to pin down. While some strains appear to be commensal or even mutualistic, others display entomopathogenic properties, making them promising candidates for pest control. For example, oral ingestion of *P. agglomerans* strain PaR38 results in 80-100% mortality in peach aphid *Myzus persicae* after 72 hours (18). Some *P. agglomerans* strains have already been developed into commercialized biocontrol products *e.g.* Bloomtime Biological, which uses strain E325 for suppressing fire blight (40–42). However, their underlying modes of action have been little explored. To determine to which extent *P. agglomerans* is a relevant candidate for use in biological control, the nature of its interaction with plants and its circulatory capacity within them are also important aspects to consider for a durable systemic protection against insect pests, but also to avoid the application of a potentially phytopathogenic bacterium on crops.

Recently, our team accidentally isolated a strain of *P. agglomerans* (named strain ELIV) during a routine colony transfer. We investigated this strain’s impact on the black bean aphid *Aphis fabae*. As we observed that ingestion caused sudden death in aphids, we conducted toxicity tests through contact and oral infection experiments. As some strains of *P. agglomerans* can grow on the surface of plants or even circulate inside them (24), we tested the ELIV strain’s ability to transit through plants and infect aphids feeding on them. We also determined the effect of *P. agglomerans* ELIV on plant growth. Finally, this strain’s genome was sequenced to identify potential virulence factors. Overall, our results indicate that *P. agglomerans* ELIV has strong entomopathogenic properties, but also slightly negatively affects plant growth without being able to circulate within the plant. Our study shows that the use of bacteria as bioinsecticides is not without risk for agriculture, as it can generate consequences for plant growth. A comprehensive approach is therefore necessary to assess the potential of bacterial biopesticides.

## 2 Materials and methods

### 2.1 Biological material and bacterial identification

#### 2.1.1 Aphid rearing

The seedlings of *Vicia faba* (cv. “Axel”) used in experiments were grown for two weeks in 6×6×5.8 cm plastic pots filled with commercial universal potting soil in controlled conditions (20 ± 1 °C, 60 ± 5% relative humidity, 16:8h light/dark photoperiod, 2.5 klux light intensity). *Aphis fabae* (clone A06-407) rearings were established from a single apterous parthenogenetic female. Clone A06–407 was initially collected from *Chenopodium album* in St. Margrethen, Switzerland, and was kindly provided by Prof. Christoph Vorburger (EAWAG: Swiss Federal Institute of Aquatic Science and Technology) (43).

#### 2.1.2 Bacterial culture and identification

The *Pantoea agglomerans* “ELIV” strain used in this study has firstly been isolated from a contaminated Petri dish using the streak plate technique (44). After isolation and 5 days of incubation at 20 °C, a single colony was collected using a sterile loop and diluted in 1 mL of PBS, the concentration being about 3.45×10^8^ CFU/mL. Morphological features were examined by light microscopy: *P. agglomerans* ELIV colonies are fast-growing, of a circular shape with entire edges, and appear yellow-orange with a glossy appearance and a viscous texture. Long-term preservation was done by freezing at -80 °C in culture medium with 20% (w/v) glycerol.

For the following experiments, a single bacterial colony was first grown to an early log phase in tryptic casein soy (TCS) broth 3% on a gyratory shaker (160 rpm) at 20 °C (45). When an optical density (OD) at 600 nm of 0.5-0.7 was reached during growth phase, the bacterial material was centrifuged (5 min, 4000 rpm, 20 °C) and washed with 20 mL of sterile phosphate-buffered saline (PBS, Sigma) solution twice, then suspended in PBS to obtain an OD of 1 at 600 nm, leading to a bacterial concentration of 4.75×10^7^ CFU/mL.

For strain identification, a single colony was collected from the Petri dish using a sterile loop and suspended in 50 µL of sterile water. Genomic DNA amplification by PCR was performed following the methodology of (46). The generalist primers used for the amplification of the 16S rRNA gene were 27_F (5’-AGAGTTTGATCCTGGCTCAG-3’) and 1492_R (5’-TACCTTGTTACGACTT-3’) as they allow the amplification of nearly the entire length of the gene (47). Samples were sent to Microsynth Seqlab GmbH (Göttingen, Germany) for Sanger sequencing. BLAST matching via the NCBI database was used to identify bacterial species.

### 2.2 Is *P. agglomerans* pathogenic to aphids?

#### 2.2.1 Oral infection assay

Due to their piercing-sucking feeding mode, aphids are mainly sensible to bacterial pathogens via ingestion (48). Thus, to test the effects of *P. agglomerans* on aphid survival, *A. fabae* individuals were fed an artificial medium containing the bacterium to enable bacterial colonization of the digestive tract (12, 49). Fifteen day-old apterous adult females of *A. fabae* were left on 2-week-old *V. faba* plants for 24 hours to produce nymphs. After the removal of adults, newborn aphid nymphs were kept on the same plants for 5 days prior to infection experiments. Third-instar aphid nymphs were then fed for 24 hours on an artificial diet prepared according to (50) and provided by Viridaxis S.A. (Charleroi, Belgium).

One hundred μL of bacterial solution (or 100 μL of sterile PBS for control treatment) were mixed with 20 mL of aphid diet, resulting in a concentration approximating 5×10^4^ CFU/mL. The diluted treatment was prepared by mixing 50 µL of bacterial solution and 50 µL of PBS in 20 mL of aphid diet, for a final concentration of 2.5×10^4^ CFU/mL. After oral infection, diagnostic PCR was performed according to (46) on randomly selected aphids to verify the bacteria’s presence in infected aphids, and absence in controls. All experiments were conducted on 10 day-old *A. fabae* individuals, either with a concentrated (5×10^4^ CFU/mL) or diluted (2.5×10^4^ CFU/mL) bacterial diet prepared as previously described. Aphids fed on a sterile diet were used as controls. For experiments examining the survival of aphid hosts, three conditions were studied: (I) control treatment, and oral infection with a (II) concentrated or (III) diluted *P. agglomerans* solution. All other experiments in this study were conducted using only the concentrated solution, thus testing two conditions: I. control treatment and II. infection with a concentrated solution of *P. agglomerans*.

After oral infection, aphid survival was monitored by placing 10 orally infected individuals (either with a diluted or concentrated bacterial solution) on a *V. faba* leaf turned over on its adaxial side and placed in a Petri dish filled with a 2 mm-high jelly agar base (0.1%). The Petri dish lid was perforated and covered with fine mesh fabric (~450 μm) to prevent condensation. The whole setup was sealed with a Parafilm^®^ M strip then flipped over so that the aphids were positioned upside down, mimicking their natural feeding position (51). All tested aphids were considered genetically identical as they were issued from the same clone and reared in the exact same conditions, thus limiting variability among replicates. During the 5 days following infection, live and dead aphids were recorded once per day for each monitoring Petri dish. Fifty replicates (10 aphids per replicate) were performed for the concentrated treatment, 38 for the diluted treatment, and 49 for controls.

#### 2.2.2 Contact pathogenicity assay

*Pantoea agglomerans* ELIV contact pathogenicity was tested by spraying 50 mL of concentrated bacterial solution with a TriggerSpray MINI1000/XMXS1000 manual sprayer directly onto 10-day-old apterous *A. fabae* individuals placed on a fine mesh sieve (~450 μm). Controls were sprayed with sterile PBS. Fifty aphids were treated for each condition. Following the spraying, insects were placed on 2-week-old *V. faba* seedlings. After 24 hours, the number of surviving aphids was recorded every 2 days for 8 days.

### 2.3 Can *P. agglomerans* infect plants and circulate within them?

#### 2.3.1 Verification of the absence of *P. agglomerans* in healthy plant tissues

Since *P. agglomerans* is pervasive in the environment, including in plants (25), we first verified the absence of *P. agglomerans* in the tissues of the *V. faba* plants used in the experiments. Sixteen randomly selected plants were unpotted and their roots were thoroughly cleaned with pure water to remove all soil residue; excess water was absorbed using sterile paper towels. From each plant, two leaf discs (from different leaves), two stem sections, and two root sections were collected using a sterile 2 cm diameter circular cutter. Each sample was placed in a clean Eppendorf tube and instantly frozen by immersion in liquid nitrogen for 3 seconds. Samples were stored at -80 °C until plant DNA extraction using the CTAB method described in (52). The absence of *P. agglomerans* was verified by performing a diagnostic PCR as described in (46), using generalist bacterial primers 27_F (5’-AGAGTTTGATCCTGGCTCAG-3’) and 1492_R (5’-TACCTTGTTACGACTT-3’), as well as *P. agglomerans*-specialist primers PANAG_infB_F (5’-GATGACGARGCCATGCTGC-3’) and PANAG_infB_R (5’-TGTCCGGCGTGCCGGCTG-3’) (53).

#### 2.3.2 Is *P. agglomerans* capable of infecting plants and the aphids feeding on them?

To determine the ability of *P. agglomerans* ELIV to infect plants and aphids feeding on them, but also to assess the bacterium’s effect on host plant growth parameters, a system for watering plants with a bacterial solution was set up by mixing 7 mL of *P. agglomerans* bacterial solution of 4.75×10^7^ CFU/mL concentration (or sterile PBS for control) in 1 L of 3% TSC Broth medium. The mixture was placed on a gyratory shaker (160 rpm) at 20 °C for 6 hours to allow bacterial growth, resulting in a solution approximating 2×10^8^ CFU/mL. Twenty-day-old *V. faba* seedlings were then evenly watered with 200 mL of the mixture. A total of 45 V*. faba* seedlings were used: 9 to test environmental acquisition by plants (2 controls, 7 treatments), 24 to study aphid infection via the host plant (12 controls, 12 treatments), and 12 to assess plant growth parameters (5 controls, 7 treatments).

To observe whether *P. agglomerans* can circulate via the phloem sap, plants were watered with a 2×10^8^ CFU/mL bacterial solution, then tested seven days later for the presence of *P. agglomerans*. Test and control plants were first removed from their substrate, and their roots were cleaned with pure water. Then, DNA extraction and PCR diagnosis of plant parts (leaf, stem, roots) were performed as described above. Simultaneously, experiments were conducted on *P. agglomerans*-watered *V. faba* seedlings to determine whether *P. agglomerans* can infect healthy aphids via the plant. One week after watering with a 2×10^8^ CFU/mL bacterial solution, eight apterous 10 day-old *A. fabae* individuals were deposited on each plant; numbers of live and dead aphids were recorded every other day for 8 days.

#### 2.3.3 Is plant growth influenced by *P. agglomerans*?

*Pantoea agglomerans* includes strains that are potential plant pathogens or endophytes with plant growth-promoting properties. We therefore studied the nature of the interaction between *V. faba* and *P. agglomerans* ELIV by evaluating plant growth parameters in aerial and root parts, *i.e.* (I) morphological and physiological parameters, and (II) mineral composition. A total of 23 parameters were measured 7 days after watering with the bacterial solution (or a sterile PBS solution for control) prepared as described above. Aerial and root parts were treated separately. Leaves and petioles were sectioned from the stem using a sterile scalpel. Roots were carefully cleaned with clean water on a stainless-steel sieve (~0.84 mm), then scanned using an EPSON Perfection V800 scanner and Epson SCAN 2 software v6.6.64.0. Root scans were imported into ImagJ v1.54g (54) and traced using Smartroot (55).

- *Morphological and physiological parameters*. For aerial parts, the following plant growth parameters were assessed: leaf dry mass, stem length, and leaf area. For the root parts, root dry mass, root surface area and maximal root length were measured. *Dry mass.* Plant dry mass is used to estimate tissue density (56). After separation, aerial and root parts were placed in a drying oven for 7 days at 60 °C, then weighed using a high-precision scale (Mettler-electrobalance Me22, sensitivity: 0.001 mg, Mettler-Toledo). *Stem length.* Plant height is a performance indicator associated with growth form, competitive vigor, fertility and potential lifespan (56, 57). Stems were placed on a white paper sheet and photographed with a Canon EOS M50 Mark II 15-45mm camera, then measured using ImagJ v1.54g (54). *Specific leaf area (SLA).* SLA, *i.e.* the leaf area/leaf dry mass ratio, is an indicator of relative plant growth rate (56). The surface area of leaves and petioles was measured using a LI-3100C Area Meter scanner. *Specific Root Length (SRL).* SRL is the length-to-mass ratio (L/M) of a root fragment and is an indicator of the plant’s soil exploitation capacities, *i.e.* the balance between resource acquisition (length) and the investment to acquire these resources (mass) (56, 58). *Roots/shoots ratio.* The ratio of root dry mass to leaf dry mass (R/S) estimates the distribution of energy within the plant. The higher this ratio, the more energy is invested in aerial parts; conversely, a low ratio indicates an investment directed towards the roots (59).
- *Mineral composition*. Carbon contents, nitrogen contents, sulfur contents and C/N ratio were assessed. Both C and N are essential for plant growth, reproduction and stress responses (60). Nitrogen contents serve as an indicator of litter quality and adequate resource acquisition (56, 57). Sulfur compounds play an important role in photosynthesis by conferring protection against inactivation due to heat and cold stress (61, 62), but also against viral development (63, 64). The C/N ratio is an indicator of nitrogen use efficiency and resource conservation (57). Analyzes were performed by the Mineral & Organic Chemical Analysis platform, UCLouvain (Louvain-la-Neuve, Belgium) using a vario EL Cube CNS elemental analyzer (Elementar) following the manufacturer’s protocol.

### 2.4 Sequencing and annotation of *P. agglomeran*s ELIV genome

#### 2.4.1 Genome sequencing and assembly

The genome of *P. agglomerants* ELIV was sequenced using a ONT GridION sequencer. Prior to GridION nanopore sequencing, DNA was extracted from one fresh colony using a Monarch Genomic DNA Purification Kit (New England Biosciences), according to the manufacturer’s protocol. Extraction purity was assessed using a NanoDrop One/OneC spectrophotometer (ThermoFischer Scientific), DNA concentration using a Qubit^®^ 3.0 Fluorometer (Invitrogen), and DNA integrity on a 1% agarose gel. The sample displayed a DNA Integrity Number of ≥ 9.5. Libraries were then prepared from 200 ng of genomic DNA using a Rapid Barcoding kit 24 V14 kit (RBK114.24), in accordance with the recommended Oxford Nanopore Technologies protocol. DNA molecules were cleaved by a transposase and barcoded tags were attached to cleaved ends. Barcoded samples were then pooled and Rapid Sequencing Adapters were added to tagged ends. Pooled libraries were sequenced on a GridION (Oxford Nanopore Technologies) using a FLO-MIN114 (R10.4.1) flow cell for a 48-hour run. Data acquisition was managed by MinKNOW 24.02.10. Basecalling was performed in real-time using the integrated Dorado 7.3.9 with the High accuracy model. Finally, long-read sequences were assembled *de novo* using Canu v2.2 (parameters: genomeSize = 5m; maxInputCoverage = 50) (65). Raw sequence reads have been deposited in the NCBI SRA database (BioProject PRJNA1430600) (66).

#### 2.4.2 Genome annotation

The genome was annotated on the MicroScope platform (67, 68). Complete macromolecular systems (flagellum and secretion systems) were predicted using MacSyFinder version 2.1.2 (69). Virulence genes were predicted using the virulence factor database (VFDB, www.mgc.ac.cn/VFs/) (70): datasets of virulence genes were downloaded and BLASTp were performed (minimum 30% aa identity).

#### 2.4.3 Phylogenomic analysis

To position *P. agglomerans* ELIV in the species’ diversity, we reconstructed a phylogenetic tree using a set of concatenated single-copy core protein sequences shared among different *P. agglomerans* representing diverse ecological niches (**Supplementary Table 1**). Single-copy orthologs were identified by running BUSCO v5.8.3 (71) with the gammaproteobacteria_odb12 lineage database, yielding 318 single-copy orthologs shared across all genomes in the dataset. Amino acid sequences of each orthologs group were aligned using MAFFT v7.526 (72), and poorly or ambiguously aligned regions were removed using TrimAl v1.5 (automated1 option) (73). Output alignments were concatenated into a supermatrix using AMAS v1 (74), treating each orthologs group as a separate partition. A Maximum Likelihood phylogenetic tree was inferred using IQ-TREE v2.0.7 (75), with ModelFinder selecting the best-fit evolutionary model for each partition based on the Bayesian Information Criterion (BIC) (76). Branch support was evaluated with 1000 ultrafast bootstrap replicates (77, 78). The resulting unrooted tree was rooted using *E. amylovora* as an outgroup and visualized with iTOL v7 (79).

### 2.5 Statistical analysis

*Impact of P. agglomerans on aphid host fitness*. Data analysis was performed using the R software v2023.09.1 + 494.pro2 (80) with *factomineR* v2.11, *rstatix* v0.7.2, *survival* v3.5.5, *coxme* v2.2-22, *car* v3.1.2, *emmeans* v1.8.6, *multcomp* v1.4.25, *lmperm* v2.1.0, and *ranger* v0.16.0. Regarding aphid host survival experiments, a Cox frailty regression model was carried out to integrate the survival proportion in each batch over time, including random effects. Treatment with concentrated bacterial solution was used as the intercept. To compare aphid mortality between the two test treatments, statistical analyses were performed for each day separately: normality of data was tested with a Shapiro test and homogeneity of variances via a Levene test. A Mann-Whitney test was hence carried out for day 1, and Student tests were performed for days 2, 3, 4, and 5. For contact pathogenicity experiments, a Cox model and a Log-rank test were carried out.

*Impact of P. agglomerans on plant growth parameters*. Comparison tests of the two conditions (control and treatment with a concentrated bacterial solution) were performed for each parameter. Data distribution was verified using a Shapiro test and homogeneity of variances was tested with a Levene test. Since the conditions of normality and homogeneity were not met, Mann-Whitney tests were carried out for the following parameters: SLA, root/shoot ratio, aerial part N contents, root part S contents. For all other plant growth parameters studied, Student tests were performed. As the sample size was limited, p-values were corrected to account for the multiplicity of parameters using the Benjamini-Hochberg method.

## 3 Results

### 3.1 Bacterial identification

BLASTn of the sequence encoding 16S rRNA indicates that the colony isolated from the contaminated Petri dish belongs to *Pantoea agglomerans*, with strain LS1 (isolated from the rhizosphere of *Sparganium erectum*) identified as the closest match (Per. Ident 99.87%).

### 3.2 *P. agglomerans* is highly pathogenic to aphids when ingested

#### 3.2.1 Oral infection assay

**Figure 1:**
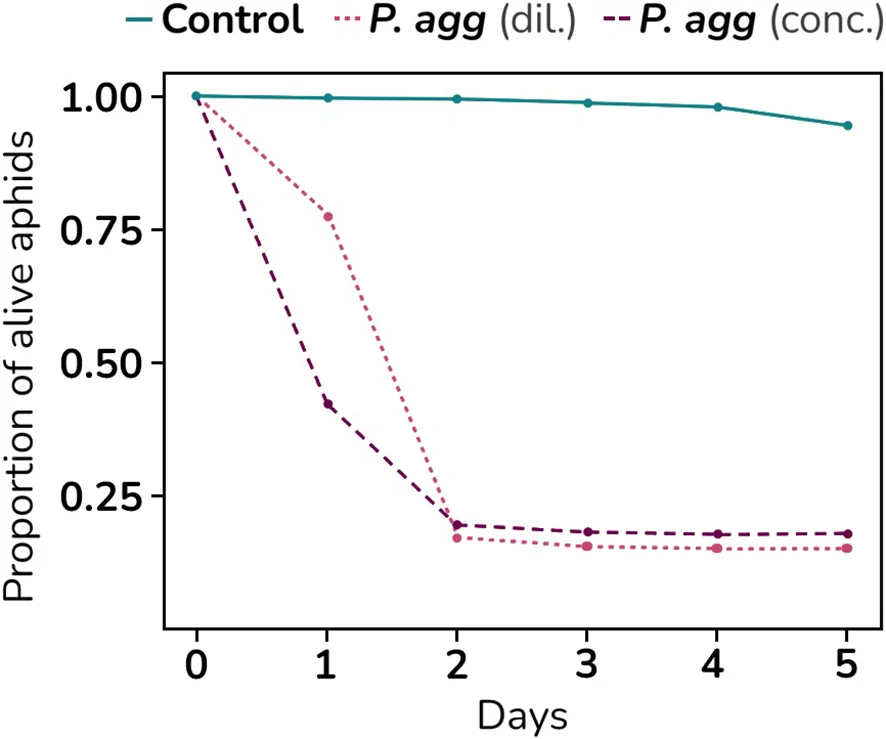
Proportion of alive *A. fabae* individuals after *P. agglomerans* (*P. agg*) ELIV oral infection, either with a concentrated (n = 50) or diluted (n = 38) bacterial solution, and control aphids (n = 49). Each replicate corresponds to a batch of 10 aphids of the same condition. Statistical comparison based on Cox frailty regression tests.

Our results show that *P. agglomerans* ELIV strikingly affects the survival of orally infected aphids; after 48 hours, less than 25% of aphids remained alive following the ingestion of bacterial solution, whether concentrated or diluted (p < 0.001, Cox frailty regression test) (**Figure 1**). Controls displayed a 70% lower risk of death compared to infected aphids (Exp(coef) = 0.305, Cox frailty regression test).

**Figure 2:**
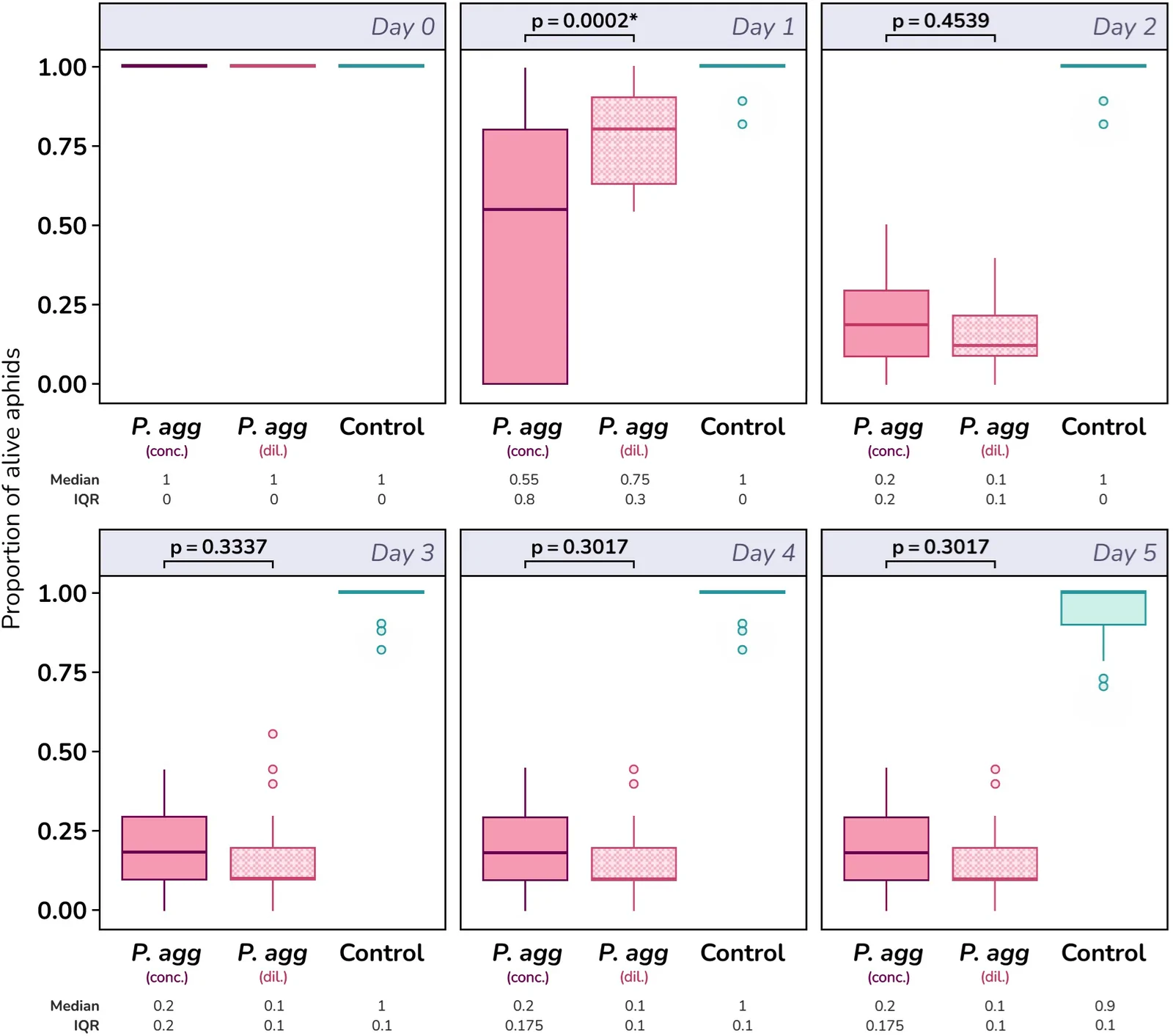
Distribution of the proportion of *A. fabae* individuals alive after *P. agglomerans* ELIV oral infection, either with a concentrated (n = 50) or diluted (n = 38) bacterial solution, and control aphids (n = 49). Each replicate corresponds to a batch of 10 aphids of the same condition. Statistical comparison based on Mann-Whitney tests for Day 1 and Student tests for all other days.

Regarding aphid mortality, no difference was observed between diluted and concentrated bacterial solutions (p = 0.953; Exp(coef) = 1.007, Cox frailty regression test). Yet, when analyzing aphid mortality rates day by day (**Figure 2**), mortality seemed to be more abrupt for the concentrated treatment, but only on day 1 post-ingestion (p < 0.001, Student test).

#### 3.2.2 Contact pathogenicity assay

**Figure 3:**
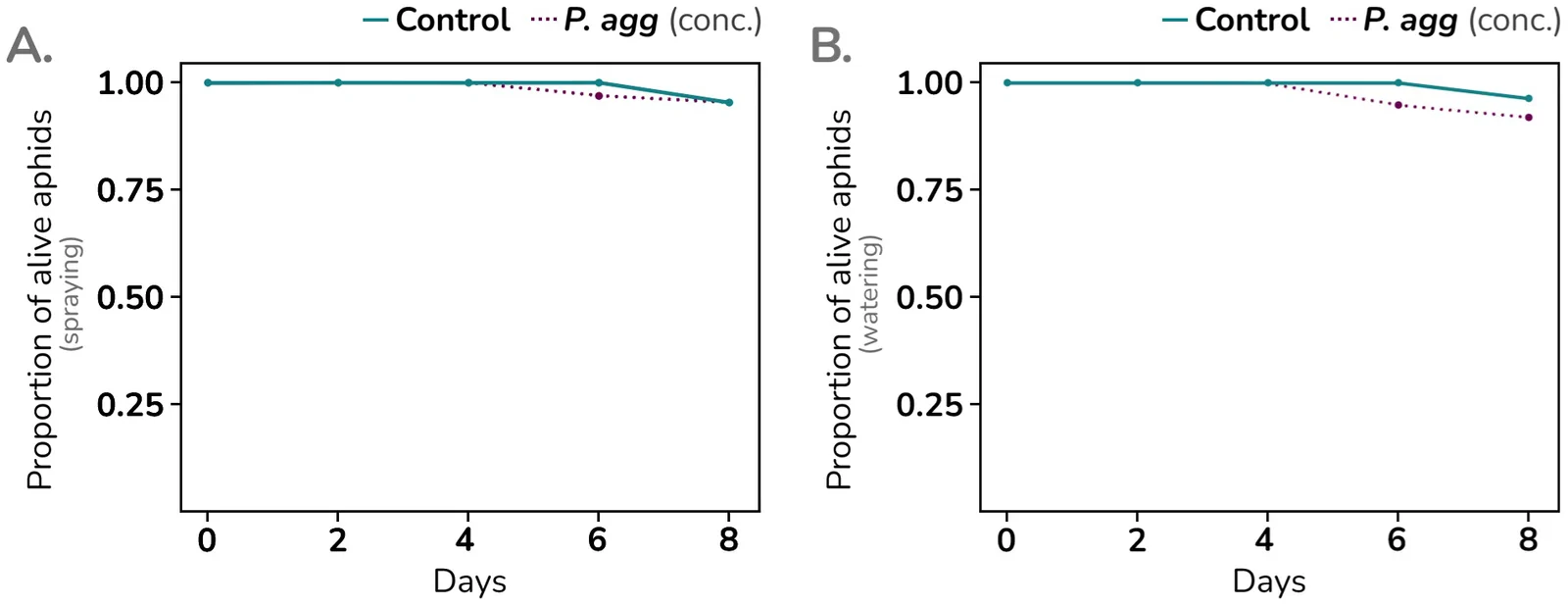
Proportion of alive *A. fabae* individuals **(A)** after being sprayed with a concentrated *P. agglomerans* ELIV solution (5×10^4^ CFU/mL) compared to controls (n = 50 for both conditions), **(B)** after being reared on *V. faba* plants watered with *P. agglomerans* ELIV (2×10^8^ CFU/mL) compared to controls (n = 12 for both conditions, each replicate corresponding to a plant hosting 8 aphids). Statistical comparisons based on Cox frailty regression tests.

Unlike the oral infection test, spraying a concentrated solution of *P. agglomerans* ELIV onto *A. fabae* individuals did not cause any toxic effects through contact; no difference in survival rate was observed between infected aphids and controls (p = 0.4, Log-rank test; p = 0.323, Cox frailty regression test) (**Figure 3A**).

### 3.3 *P. agglomerans* infects plants but does not circulate in the vascular system

#### 3.3.1 *P. agglomerans* ELIV is only found in roots after watering

Diagnostic PCR results confirmed the absence of *P. agglomerans* ELIV in the leaves, stems, and roots of healthy *V. faba* seedlings prior to experiments. Environmental acquisition assays showed that after watering plants with a 2×10^8^ CFU/mL *P. agglomerans* ELIV solution, the bacterium was detected only in root parts. PCR gel electrophoresis results can be viewed in the **Supplementary Material** section (**Supplementary Figure 1**).

#### 3.3.2 *P. agglomerans* does not infect aphids feeding on infected plants

*Pantoea agglomerans* did not infect aphids feeding via the plant; no difference in mortality was observed between *A. fabae* individuals reared on *P. agglomerans* ELIV-treated plants and controls (p = 0.3, Log-rank test; p = 0.187, Cox frailty regression test) (**Figure 3B**). Diagnostic PCR confirmed that neither aphids reared on a treated plant nor controls were infected with the bacterium.

#### 3.3.3 *P. agglomerans* slightly influences plant growth

Among the 23 studied plant growth parameters, no significant differences were observed between *P. agglomerans* ELIV-treated plants and controls regarding aerial parts, stems and roots parts dry masses, stem length, leaf surface area, SLA, SRL, root/shoot ratio, root length and surface area, C, N, S contents in aerial and root parts separately, nor the C/N ratio in aerial parts (**Table 1**). Treated plants displayed a lower total leaf dry mass (p < 0.001, Student test) compared to controls, and a reduction in total C contents (p = 0.036, Student test), C/N in aerial parts (p = 0.033, Student test), and total C/N (p = 0.023, Student test). However, after correcting the p-values using the Benjamini-Hochberg procedure, only the total leaf dry mass was significantly lower in *P. agglomerans* ELIV-treated plants compared to controls (p=0.0115, Student test with Benjamini-Hochberg correction).

**Table 1:** Evaluation of plant growth parameters (median values ± IQR) of *V. fabae* plantlets 7 days after being watered with a *P. agglomerans* ELIV solution.

Plant health parameter	Control (± IQR)	*P. agglomerans* treatment (± IQR)	p-value	p-value (corrected)
Aerial parts dry mass *(mg)*	915.6 ± 67.9	724.8 ± 116.8	0.1245	0.3182
Stem dry mass *(mg)*	361.9 ± 56.7	337.3 ± 146.4	0.5186	0.7455
**Leaf dry mass *(mg)***	**553.7 ± 67.6**	**387.5 ± 41.95**	0.0005*	0.0115*
Root parts dry mass *(mg)*	144.8 ± 50.5	109.8 ± 025.1	0.2171	0.4161
Stem length *(mm)*	570 ± 92.41	530.53 ± 102.44	0.1509	0.3155
Leaf surface area *(mm²)*	2725 ± 69	1865 ± 505	0.1298	0.2985
SLA *(mm^2^·mg^-1^)*	5.25 ± 0.69	4.58 ± 1.22	0.5303	0.7175
Maximum root length *(mm)*	962.23 ± 106.1	877.8 ± 181.65	0.7564	0.7908
Root surface area *(mm²)*	22216.68 ± 7712.49	20058.5 ± 4320.15	0.2273	0.4021
SRL *(m·g^-1^)*	6.57 ± 3.99	7.88 ± 4.43	0.3901	0.6409
Root/shoot ratio	0.26 ± 0.09	0.27 ± 0.13	0.5303	0.7175
C contents in aerial parts *(%)*	42.58 ± 0.66	42.16 ± 1.37	0.6372	0.8142
C contents in root parts *(%)*	48.99 ± 1.04	47.6 ± 1.54	0.0929	0.3561
**Total C contents *(%)***	**45.73 ± 0.24**	**44.89 ± 1.42**	0.0366*	0.2105
N contents in aerial parts *(%)*	6.13 ± 0.34	6.67 ± 0.91	0.1061	0.3486
N contents in root parts *(%)*	5.18 ± 0.18	4.76 ± 0.455	0.4288	0.6575
Total N contents *(%)*	5.51 ± 0.2	5.7 ± 0.31	0.1121	0.3223
S contents in aerial parts *(%)*	0.6 ± 0.07	0.62 ± 0.1	0.7100	0.8165
S contents in root parts *(%)*	0.44 ± 0.01	0.4 ± 0.015	0.0695	0.3197
Total S contents *(%)*	0.52 ± 0.04	0.55 ± 0.07	0.7404	0.8109
**C/N *(aerial parts)***	**6.94 ± 0.57**	**6.32 ± 0.59**	0.0332*	0.2545
C/N *(root parts)*	9.71 ± 0.44	10 ± 0.715	0.7030	0.8510
**Total C/N**	**8.16 ± 0.5**	**7.9 ± 0.67**	0.0227*	0.2611

n = 5 for controls, n = 7 for treatment. Statistical comparison based on Student and Mann-Whitney tests. Correction of p-values was assessed using the Benjamini-Hochberg method. * represents significant p-values (p < 0.05). Bold values represent plant growth parameters that yielded significant p-values.

### 3.4 Genomic and phylogenetic analyses

#### 3.4.1 General genomic features of *P. agglomerans* ELIV

**Figure 4:**
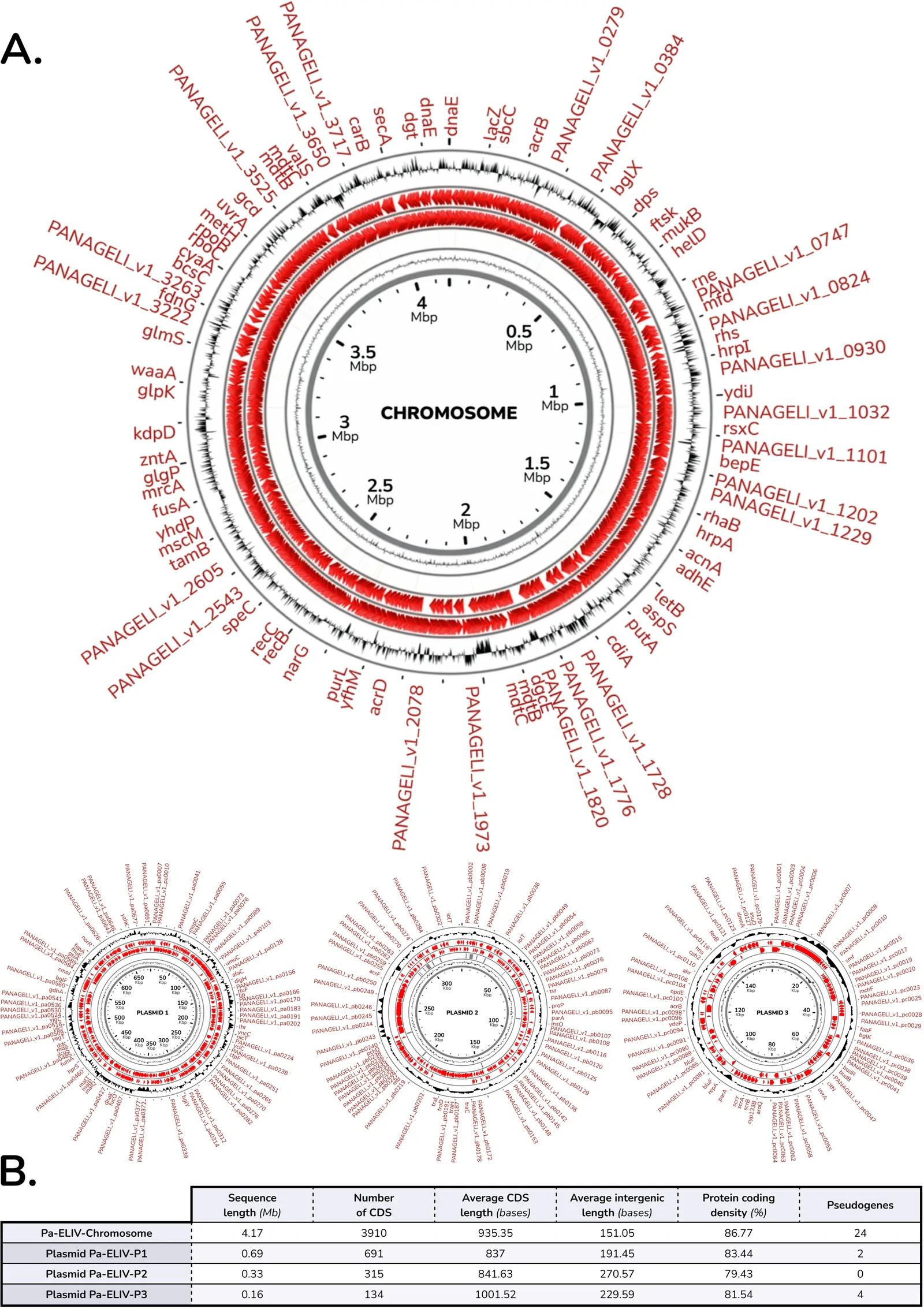
Genome sequencing of *P. agglomerans* ELIV. **(A)** Circular genomic plots of the bacterial strain’s chromosome and three plasmids **(B)** Sequence length, number and average length of coding DNA sequences (CDS), average intergenic length, protein coding density and number of pseudogenes of *P. agglomerans* ELIV.

A total of 238, 000 reads were sequenced for a total of 2, 622 Mb, representing an average fragment size of 11 kb and a coverage of 490x. Sequencing and assembly enabled the generation of the complete genome of *P. agglomerans* ELIV (5.35 Mb), consisting of a circular chromosome of 4.17 Mb and three plasmids (0.69, 0.33, and 0.16 Mb) (**Figure 4**) Genome assembly is highly complete, with 99.1% of the Benchmarking Universal Single-Copy Orthologs (BUSCO) genes represented against the gammaproteobacteria database. A total of 96.5% of these genes are single copy, 2.6% are duplicated, 0.6% are fragmented, and 0.3% are missing. A total of 5, 050 CDS (coding DNA sequences) were identified. The assembly and annotation are available on GenBank under the following accession number: JBVQST000000000 (BioProject PRJNA1430600).

#### 3.4.2 Phylogenetic positioning of *P. agglomerans* in the *Pantoea* diversity

**Figure 5:**
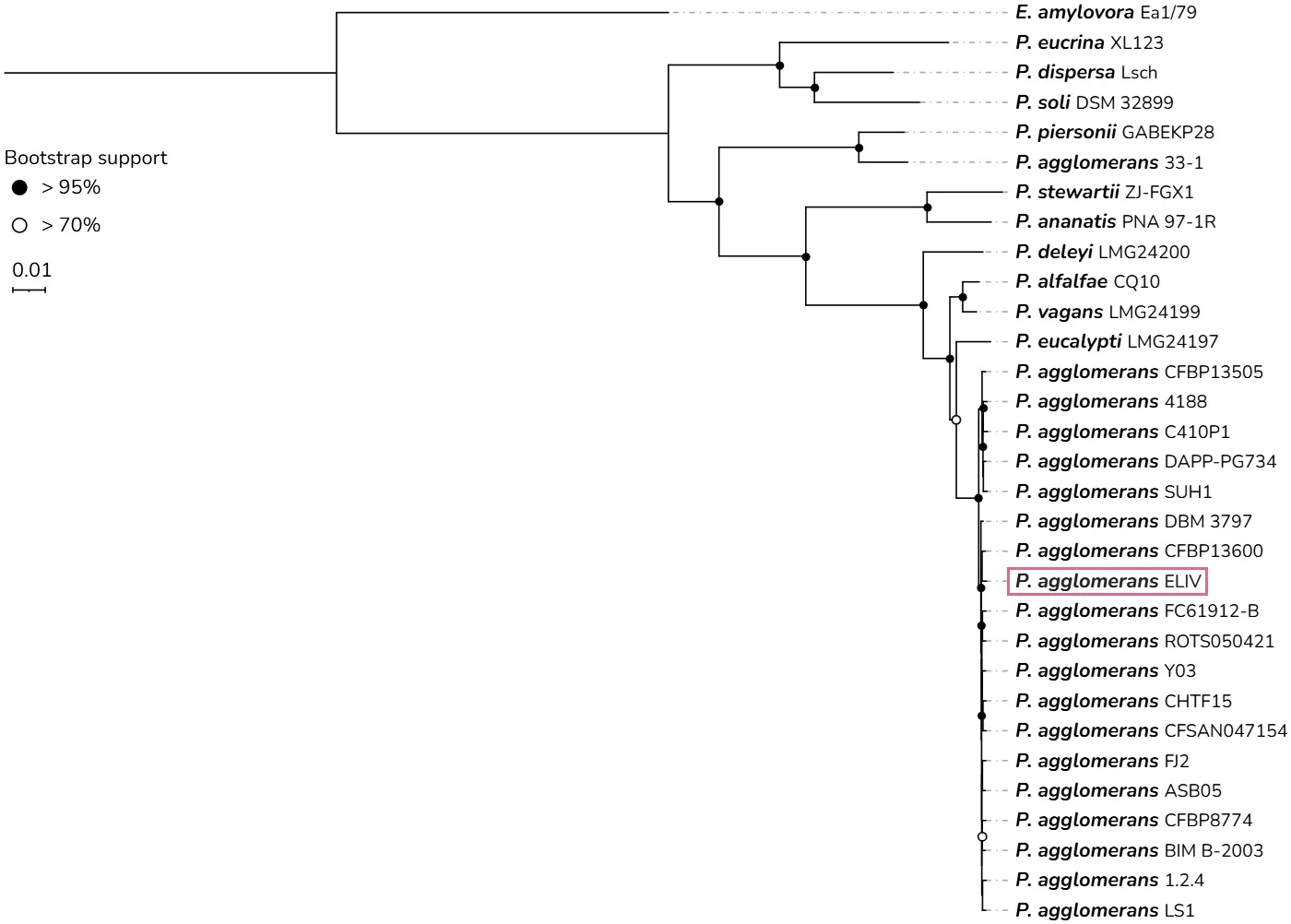
Maximum likelihood (ML) phylogeny based on a concatenated alignment of 318 single-copy orthologs revealing the phylogenetic placement of the *P. agglomerans* ELIV strain relative to other members of the species and the *Pantoea* genus. Node coloration reflects bootstrap support. The tree was rooted by using *Erwinia amylovora* Ea1/79 as an outgroup.

Maximum likelihood (ML) phylogeny based on a concatenated alignment of 318 single-copy orthologs reveals the phylogenetic position of the *P. agglomerans* ELIV relative to other members of the species and genus *Pantoea* (**Figure 5**). *Pantoea agglomerans* ELIV is phylogenetically closest to strain CFBP13600 isolated from seeds of common bean *Phaseolus vulgaris*.

#### 3.4.3 The secretion systems and the array of virulence factors encoded by the *P. agglomerans* ELIV genome

Regarding the macromolecular systems encoded by *P. agglomerans* ELIV (**Supplementary Table 2**), genome annotation suggests that the strain is capable of expressing a complete flagellum. Moreover, the strain can express a type III secretion system (T3SS) and several type I (T1SS) and type V (T5aSS and T5bSS) secretion systems. The genes encoding type I and V secretion systems are distributed across the chromosome and plasmids. Results also indicate that the strain can express a type VI secretion system (T6SS) and a type IV secretion system (type F).

Annotation shows that *P. agglomerans* ELIV is capable of expressing a wide range of virulence factors (**Supplementary Table 4**), many of these being part of iron acquisition systems. Furthermore, AntiSMASH prediction (**Supplementary Table 3**) suggests that *P. agglomerans* ELIV can produce enterobactin, deferoxamine E (DFOE), and enantio-pyocheline, three types of siderophores used to capture iron in their environment, but also aryl polyene (APE) which is linked to increased biofilm formation. Although very few direct toxins have been identified, our results show that *P. agglomerans* ELIV possesses a rich virulome.

## 4 Discussion

Aphid pest control mainly relies on synthetic insecticides (81); one promising alternative is the use of entomopathogenic microbes to control aphid populations. We isolated a new *P. agglomerans* (ELIV) strain exhibiting high pathogenicity against *A. fabae*, a major crop pest in temperate zones. Our results confirm that the species *P. agglomerans* is a reservoir of aphid-killing bacteria.

Oral infection assays demonstrated that *P. agglomerans* ELIV kills three-quarters of aphids 48 hours after ingestion. Some bacteria with entomopathogenic properties against aphids have already been identified, such as *Serratia marcescens* Db11 (12), *Escherichia coli* K12 (82), and epiphytic strains of *Pseudomonas syringae* with susceptibility varying among aphid species (83). Paliwal et al. (18) successfully identified a set of aphid-killing bacteria from the *Pantoea* (including *P. agglomerans*) and *Pseudomonas* genera as promising candidates for aphid control; our results are consistent with those reported on *Myzus persicae* (18). Feeding aphids a concentrated solution of *P. agglomerans* ELIV halves the proportion of alive individuals on day 1, while it remains above 75% with the diluted solution. However, observed mortality is similar for both tested concentrations on day 2 (less than 25% alive aphids), suggesting a delayed lethality for the diluted solution, likely due to the bacterial multiplication before reaching the lethal concentration. Overall, *P. agglomerans* displays strong entomopathogenic effects, making this strain an interesting candidate for controlling insect pests.

Although *P. agglomerans* ELIV demonstrates aphid-killing abilities when ingested orally, no toxic effect by contact was observed. This indicates that regarding practical applications, *P. agglomerans* ELIV cannot be applied by spraying, a common method employed in the field. Foliar application of aphid-killing bacteria has been previously observed to slow aphid multiplication on plants (18), but these results were reported for *Pseudomonas fluorescens* PpR24, with no tests being conducted with *P. agglomerans*. This decrease was possibly not observed because our study lasted 8 days compared to 20 days in (18). Aphids are piercing-sucking insects, which could limit bacterial oral assimilation and therefore the effectiveness of a spraying approach (48, 84). Although aphids lack several genes that are crucial for defense against bacterial pathogens (85), aphicidal effects are mostly observed following oral infection rather than topical application, as ingestion allows bacterial colonization of the digestive tract and *in situ* toxin production (48, 49, 86). Hence, the spraying of *P. agglomerans* ELIV could be more effective on chewing insects *e.g.* Lepidoptera caterpillars and beetles, for which the oral route is more readily accessible to bacteria. This method of application has been observed to be effective for *B. thuringiensis* var. kurstaki against foliage-feeding caterpillars, and *B. thuringiensis* var. tenebrinos for the control of Colorado potato beetle larvae and elm leaf beetle control (87). Still, the modes of action of *P. agglomerans* and *B. thuringiensis* might differ; assays must be carried out using the ELIV strain against chewing insects before drawing any conclusions.

*Pantoea agglomerans* is ubiquitous; while this species includes potentially phytopathogenic strains, it is frequently described as a plant associate endowed with plant growth-promoting properties (25, 31–33). As the isolation of *P. agglomerans* ELIV resulted from contamination during a routine transfer, the original source host could not be identified. However, phylogenomic analyses show that *P. agglomerans* ELIV is closely related to CFBP13600 strains isolated from common bean *Phaseolus vulgaris* seeds, suggesting that *P. agglomerans* ELIV is a putative rhizobacterium associated with plants, leading us to examine the interactions between *P. agglomerans* ELIV and *V. faba* plants.

While *P. agglomerans* can colonize different plant parts including leaves (28, 88, 89), its ability to circulate within plants has not been tested. Some epiphytic strains could originate from seeds during development (90), since *P. agglomerans* is a seed endophyte of numerous plant species, although airborne transmission is also conceivable (88, 91). Some bacteria can readily circulate in the xylem and the phloem; for instance, several *S. symbiotica* strains can rapidly colonize plants from the roots and infect aphids feeding on leaves (49, 59); these strains are moderately pathogenic to aphids and decrease their fitness. According to our observations, *P. agglomerans* ELIV does not appear to be able to circulate within the plant and ultimately infect aphids. Watering plants with a *P. agglomerans* solution did not result in the bacterium’s presence in stems nor leaves; diagnostic PCR confirmed its presence in root parts only. No difference in aphid survival was observed between *P. agglomerans* ELIV-treated plants and controls. However, as PCR analyses were performed at a single time point and absence of detection does not necessarily indicate absence of bacteria, these results must be treated cautiously and further experiments should be carried on several time points post-infection to help strengthen this conclusion. Moreover, localization of the bacteria within plant tissues using fluorescence *in situ* hybridization (FISH) techniques and quantitative approaches such as qPCR would allow a more comprehensive evaluation of *P. agglomerans* ELIV’s ability to circulate in plants.

Although many bacterial pesticides are now entering the market, their effects on plants are rarely assessed. This is nevertheless an important aspect to consider, as some entomopathogenic bacteria could have repercussions on plant growth, or at least interact with them in ambiguous ways. We thus investigated the nature of the interaction between *P. agglomerans* ELIV and *V. faba* by assessing various plant growth parameters. Our results show that this bacterium significantly affects leaf dry mass only, meaning that the bacterium has a negative, albeit moderate, impact on plant growth under our tested conditions. However, these results should be interpreted with caution as sample size is limited. *Pantoea agglomerans* is frequently isolated from healthy and diseased plants, animals and humans; while it is rarely described as a plant pathogen, a few strains have been found to induce leaf, stem or rot diseases in cotton, maize and *Araceae* plants (27, 92, 93), or to hinder growth in alfalfa and rice (30, 94–96). Still, few studies have looked into exact mechanisms involved in the pathogenicity of *P. agglomerans* in plants, leaving them largely underexplored. On the other hand, many *P. agglomerans* strains are known to be epiphytic or endophytic mutualists in plants, providing protection against pathogens or promoting their growth (25, 31–33, 97). Indeed, some strains are capable of inducing plant systemic resistance, such as *P. agglomerans* E278Ar in radish plants against the agent of bacterial leaf spot *Xanthomonas campestris* pv. *armoraciae* (98–100). The exopolysaccharides of several *P. agglomerans* strains can induce a form of resistance against disease in monocots, such as wheat and rice, by priming their cells to potentiate the oxidative burst immune response, which manifests as a rapid release of H_2_O_2_ following plant stress (101, 102). Some plant growth-promoting strains, such as *P. agglomerans* 33.1, have also been genetically modified with the pJTT vector carrying the cry1Ac7 gene to control the sugarcane borer *Diatraea saccharalis* (99). Again, as the effects of *P. agglomerans* are very strain-dependent (25, 33), more research is needed to better our understanding of *P. agglomerans* ELIV’s relationship with plants. For example, the use of molecular tools similar to qPFD (103) could allow to investigate if the ELIV strain is capable of eliciting plant defenses by stimulating genes involved with plant resistance mechanisms (104, 105).

To better understand the modes of action underlying *P. agglomerans* ELIV’s entomopathogenicity, we sequenced and annotated its genome. This strain possesses an arsenal of virulence factors. It is capable of expressing a complete flagellum, an essential apparatus for reaching infection sites and often necessary for adhesion to surfaces, the first step in the formation of protective biofilms (106). The repertoire of virulence factors includes numerous genes involved in chemotaxis (*e.g*. *cheA*, *cheB*, *cheR*, *cheW*, *cheY* and *cheZ*) and quorum sensing (*e.g*. *sdiA* and *luxS*), behaviors that optimize colonization of infection sites (107–109). *Pantoea agglomerans* ELIV is capable of expressing various secretory systems relevant for the delivery of toxins, effectors, and adhesins to host cells, thus contributing to the evasion of host immune responses. These secretion systems are key players for insect infection, as shown for some Gram-negative entomopathogenic bacteria *e.g. Photorhabdus*, *Xenorhabdus*, *Serratia*, *Yersinia*, and *Pseudomonas* species (110). *In silico* analysis suggests that *P. agglomerans* ELIV is capable of expressing type I (T1SS), type III (T3SS), type IV (T4SS), type V (T5SS), and type VI (TVSS) secretion systems. The type I (T1SS) secretion system facilitates the secretion of exotoxins, such as hemolysin A (HlyA), to damage host tissues (111). The type III (T3SS) secretion system is used to secrete effector proteins, such as Yop effectors, into host cells to promote virulence and colonization. It has been previously reported that *Pantoea* bacteria can use T3SS for infecting both plants and insects (112). Interestingly, *P. agglomerans* ELIV appears to be capable of expressing a T6SS, which is a contractile, bacteriophage-tail-like nanomachine used by many Gram-negative bacteria to inject toxic effector proteins into neighboring prokaryotic or eukaryotic cells (113). It is an effective weapon that *P. agglomerans* ELIV could use in the context of inter-bacterial competition and niche colonization. Our analyses identified few toxin-encoding genes in the genome, but an impressive set of genes involved in iron uptake systems, including the siderophores enterobactin, yersiniabactin, mycobactin, pyoverdine and pyochelin. Bacterial siderophores play a crucial role in insect colonization by enabling bacteria to acquire essential iron, which is often present in very limited quantities in the host. The chelation of available iron (Fe^3+^) is vital for bacterial growth, metabolic activity, and rapid proliferation. Furthermore, siderophores can chelate other metals. Our results suggest that the high pathogenicity of *P. agglomerans* ELIV could be explained by the bacterium’s predisposition to produce siderophores that compete with host iron-binding proteins *e.g.* transferrin (114, 115). In addition, siderophores may be involved in biofilm formation, enabling stable colonization. It is possible that the mode of action of *P. agglomerans* ELIV involves gut colonization leading to sepsis due to uncontrolled systemic bacterial proliferation, or to mechanical interference: that is, aphid mortality would result from physical obstruction rather than toxin-induced cytolysis or metabolic disruption (48, 84). The latter mechanism has been observed in related species of the genus *Pantoea* such as *P. aphidicola* and *P. stewartii*, whose aphid-killing properties can be attributed to transmembrane proteins that promote bacterial cell aggregation within the aphid gut, thus leading to physical blockage, impaired feeding, and eventually aphid death (48, 83, 116). Taken together, these results suggest that *P. agglomerans* ELIV is a highly competitive bacterium, capable of using various mechanisms to colonize insects, with a propensity for rapid multiplication, competition for iron, and biofilm formation. However, further experiments such as gene expression profiling or mutational studies must be performed to unveil *P. agglomerans* ELIV’s actual modes of action.

*Pantoea agglomerans* is a species whose effects are highly diverse and strain-dependent (25, 33), with some strains being reported as human pathogens and allergens (22, 26). Wound infection with *P. agglomerans* may follow the piercing or laceration of skin with thorns, splinters or other plant material, usually during performing outdoor activities such as gardening, playing or agricultural labor (22, 25, 26). Septic arthritis, synovitis or endophthalmitis are common clinical outcomes of infections with *P. agglomerans* (22). Moreover, a few *P. agglomerans* strains have been identified as phytopathogens affecting various agricultural crops (22, 27, 117), while some others are used in biological control of plant diseases (20, 40). Hence, more research is needed regarding biological risks such as the potential impact of *P. agglomerans* ELIV on human health, but also on non-target organism risks and environmental release. Indeed, an uninformed use of a biocontrol agent may affect a diverse range of non-target species such as non-pest insects, beneficial soil organisms, birds and other animals, leading to global biodiversity loss and the disruption of agroecosystems (118).

In conclusion, our study shows that *P. agglomerans* deserves special attention for insect pest control due to its powerful entomopathogenic effects; however, this bacterium can exhibit an ambiguous interaction with the plant. While many *P. agglomerans* strains are associated with plant growth-promoting (PGP) properties, *P. agglomerans* ELIV seems to have neutral to negative effects on the plant, making it not optimal for application on crops. This is an important aspect to consider when formulating bacterial bioinsecticides. The lack of contact pathogenicity towards aphids also limits the use of the bacterium in a spray application. On the other hand, its application to other types of pests, *e.g.* chewing insects, may be more promising. Our study also highlights the importance of a comprehensive approach when assessing the quality of bacteria for use in crop pest control. The case of *P. agglomerans* illustrates the value of using environmental bacteria, particularly those proliferating in the rhizosphere, as a reservoir for the development of new bacterial bioinsecticides. Ideal candidate bacteria must have potent entomopathogenic properties, be mutualistic to the plant, and be able to circulate within the plant to provide long-lasting protection against pests.

## Data Availability

The original contributions presented in the study are publicly available. This data can be found here: NCBI SRA database, accession number PRJNA1430600 (BioProject, Université Catholique de Louvain 2026).
