# Supplementary material for: Traits analysis of an aphid-killing Pantoea agglomerans strain for use in biological control
Source: Front Insect Sci. 2026 Aug 14;6:1896449. doi: 10.3389/finsc.2026.1896449 (PMC13522146; doi:10.3389/finsc.2026.1896449)
Supplement: Supplementary file 1 [file DataSheet1.pdf]

Supplementary materials for:

Traits Analysis of an Aphid-Killing *Pantoea agglomerans* Strain for Use in Biological Control

Alisa Hamidović[1]*, Nell Ghisdal[1], Linda Dhondt[1], Inès Pons[2], Thomas Dagbert[3], Mey Jerbi[1], Jérôme Ambroise[4], Bertrand Bearzatto[4], Jean-Luc Gala[4], Thierry Hance[1], François Renoz[1]

[1] Biodiversity Research Centre, Earth and Life Institute, UCLouvain, 1348 Louvain-la-Neuve, Belgium

[2] Mutualisms Research Group, Max Planck Institute for Biology, 72076 Tübingen, Germany

[3] PEPA Lab: Plant Ecology, ecoPhysiology and sustainable Agriculture, Earth and Life Institute, UCLouvain, 1348 Louvain-la-Neuve, Belgium

[4] Center for Applied Molecular Technologies, Institute of Experimental and Clinical Research, UCLouvain, 1200 Woluwe-Saint-Lambert, Belgium

* Corresponding author: Alisa Hamidović (alisa.hamidovic@uclouvain.be)

ORCID: 0009-0007-7203-6263

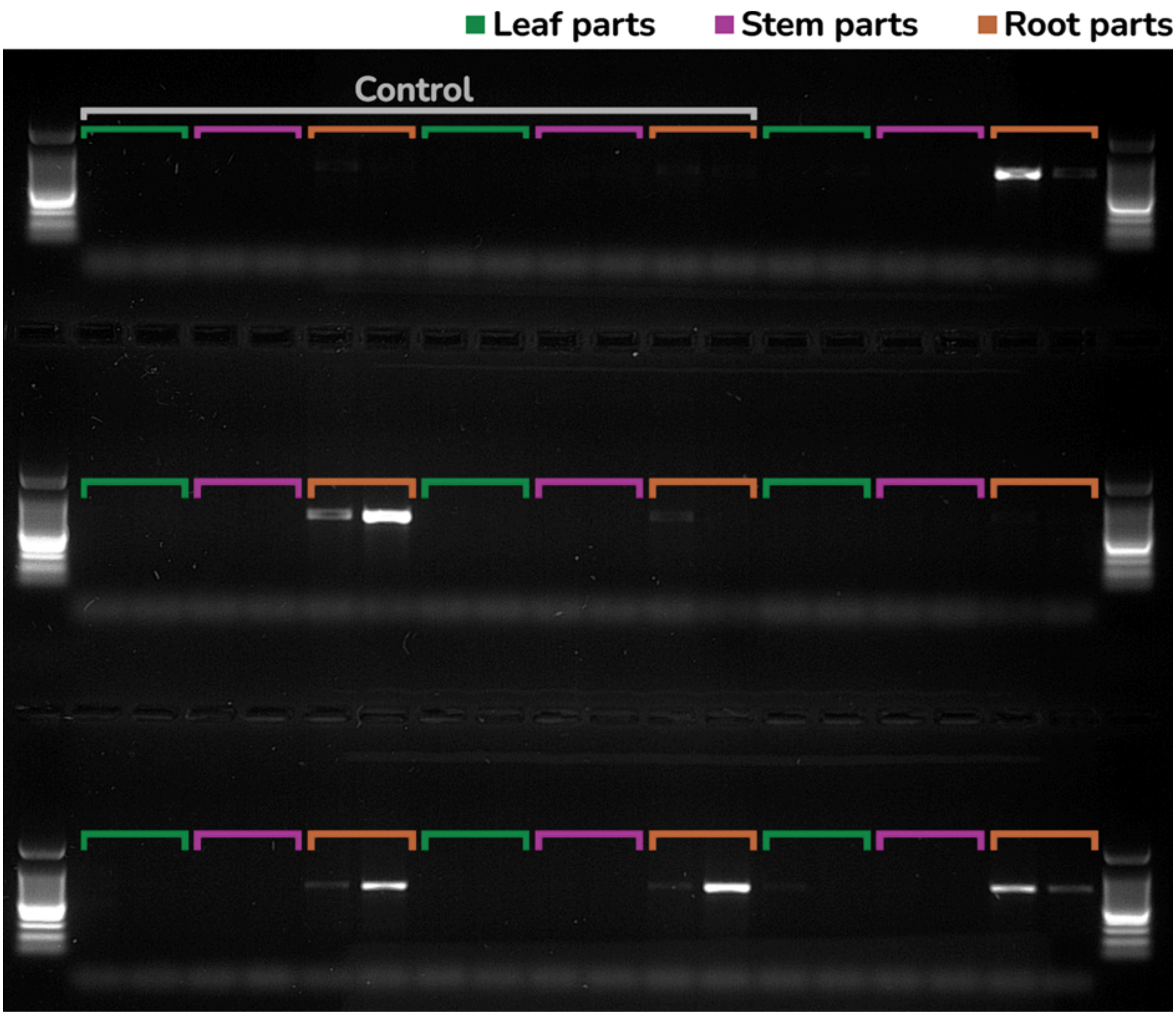

Figure S1: Diagnostic PCR performed on nine *Vicia faba* seedlings watered with a *Pantoea agglomerans* ELIV solution. Leaf parts, stem parts and root parts were diagnosed using specialist primers PANAG-infB-fwd and PANAG-infB-rev. Electrophoresis results show that only the root parts were infected.

Table S1: Accession numbers and details of the bacterial strains used for the multigene phylogenetic reconstruction of *Pantoea agglomerans* ELIV. The *Erwinia amylovora* Ea1/79 strain (green) is used as an outgroup.

Species	Strain	Assembly level	RefSeq annotation number	Isolation source	Host
Erwinia amylovora	Ea1/79	Complete (chromosome)	GCF_040125995.1	*Malus domestica*	Plant
Pantoea agglomerans	ELIV	Complete (chromosome)	NA	Contaminated Petri dish	Unknown
Pantoea agglomerans	DAPP-PG734	Complete (chromosome)	GCF_943184895.1	Olive knot caused by *Pseudomonas savastanoi pv. savastanoi*	Plant
Pantoea agglomerans	LS1	Complete (chromosome)	GCF_050916325.1	Rhizosphere of Sparganium erectum	Plant
Pantoea agglomerans	CFBP8774	Complete (chromosome)	GCF_014841465.2	*Brassica napus* seed	Plant
Pantoea agglomerans	SUH1	Complete (chromosome)	GCF_031888365.1	*Allium cepa*	Plant
Pantoea agglomerans	C410P1	Complete (chromosome)	GCF_001709315.1	Lettuce	Plant
Pantoea agglomerans	FJ2	Complete (chromosome)	GCF_038657925.1	Tobacco leaf	Plant
Pantoea agglomerans	DBM 3797	Complete (chromosome)	GCF_020783495.1	*Humulus lupulus*	Plant
Pantoea agglomerans	33-1	Complete (chromosome)	GCF_020149765.1	*Eucalyptus spp.*	Plant
Pantoea agglomerans	CFBP13505	Complete (chromosome)	GCF_005233495.2	*Raphanus sativus* cv. "Flamboyant 5"	Plant
Pantoea agglomerans	ROTS050421	Complete (chromosome)	GCF_031889005.1	*Rubus sp.*	Plant
Pantoea agglomerans	BIM B-2003	Complete (chromosome)	GCF_035582725.1	*Sphagnum fuscum* hydroalcoholic extract	Plant
Pantoea agglomerans	CFBP13600	Complete (chromosome)	GCF_014842695.2	*Phaseolus vulgaris* seed	Plant
Pantoea agglomerans	CFSAN047154	Complete (chromosome)	GCF_004117135.1	*Malus domestica cv. "Rome"* leaf	Plant
Pantoea agglomerans	FC61912-B	Complete (chromosome)	GCF_031889865.1	Stream water adjacent to a crop field	Environment
Pantoea agglomerans	Y03	Complete (chromosome)	GCF_036549535.1	Sea mud	Environment
Pantoea agglomerans	CHTF15	Complete (chromosome)	GCF_024799805.1	Diseased walnut leaves	Plant
Pantoea agglomerans	ASB05	Complete (chromosome)	GCF_009762725.1	Cherry	Plant
Pantoea agglomerans	4188	Complete (chromosome)	GCF_001662025.2	*Beta vulgaris*	Plant

Pantoea agglomerans	1.2.4	Complete (chromosome)	GCF_032200625.1	Fermented cucumber brine	Plant
Pantoea alfalfae	CQ10	Complete (chromosome)	GCF_019880205.1	Alfalfa seeds	Plant
Pantoea ananatis	PNA 97-1R	Complete (chromosome)	GCF_002952035.2	*Allium cepa*	Plant
Pantoea deleyi	LMG24200	Complete (chromosome)	GCF_022647325.1	Fungus	Fungus
Pantoea dispersa	Lsch	Complete (chromosome)	GCF_019890955.1	Drinking water	Environment
Pantoea eucalypti	LGM24197	Complete (chromosome)	GCF_009646115.1	*Eucalyptus sp.*	Plant
Pantoea eucrina	XL123	Complete (chromosome)	GCF_020079945.1	Cucumber rhizosphere	Plant
Pantoea piersonii	GABEKP28	Complete (chromosome)	GCF_027570015.1	*Homo sapiens* saliva	Human
Pantoea soli	DSM 32899	Complete (chromosome)	GCF_007833795.1	Ground	Environment
Pantoea stewartii	ZJ-FGZX1	Complete (chromosome)	GCF_011044475.1	*Dracanea sanderiana* leaf	Plant
Pantoea vagans	LMG24199	Complete (chromosome)	GCF_004792415.1	*Eucalyptus sp.*	Plant

Table S2: Prediction of macromolecular systems for *P. agglomerans* ELIV (MacSyFinder).

Macromolecular system	System type	Replicon name	Replicon type	Begin	End	Locus type	System wholeness	System score	Mandatory genes present	Accessory genes present	Number of mandatory genes present	Number of accessory genes present
Flagellum_1	Flagellum	Pa-ELI-Chromosome	Chromosome	711938	1651046	Multi_loci	1	11	Flg_fliE, Flg_sctN_FLG, Flg_sctV_FLG, Flg_flgC, Flg_sctU_FLG, Flg_sctR_FLG, Flg_sctJ_FLG, Flg_sctS_FLG, Flg_flgB, Flg_sctQ_FLG, Flg_sctT_FLG		12	0
T5bSS_16	T5bSS	Pa-ELI-Chromosome	Chromosome	749287	751005	Single_locus	1	1	T5bSS_translocator		1	0
T6SSi_18	T6SSi	Pa-ELI-Chromosome	Chromosome	830666	868048	Single_locus	0.857	12	T6SSi_tssJ, T6SSi_tssG, T6SSi_tssM, T6SSi_tssD, T6SSi_tssK, T6SSi_evpJ, T6SSi_tssH, T6SSi_tssL, T6SSi_tssE, T6SSi_tssF, T6SSi_tssI, T6SSi_tssB		12	0
T3SS_3	T3SS	Pa-ELI-Chromosome	Chromosome	890472	904486	Single_locus	1	9	T3SS_sctC, T3SS_sctJ, T3SS_sctT, T3SS_sctV, T3SS_sctQ, T3SS_sctU, T3SS_sctS, T3SS_sctN, T3SS_sctR		9	0

T5bSS_17	T5bSS	Pa-ELI-Chromosome	Chromosome	1683867	1685534	Single_locus	1	1	T5bSS_translocator		1	0
MOB_2	MOB	Pa-ELI-Chromosome	Chromosome	1981681	1982922	Single_locus	0.333	0.56	T4SS_MOBP1		1	0
MOB_1	MOB	Pa-ELI-Chromosome	Chromosome	1981681	3828612	Single_locus	0.667	0.96	T4SS_MOBP1	T4SS_t4cp2	1	1
T1SS_2	T1SS	Pa-ELI-Chromosome	Chromosome	2017287	2021978	Single_locus	1	3	T1SS_abc, T1SS_mfp, T1SS_omf		3	0
T6SSi_19	T6SSi	Pa-ELI-Chromosome	Chromosome	2146768	2181868	Single_locus	0.929	13	T6SSi_tssJ, T6SSi_tssG, T6SSi_tssM, T6SSi_evpJ, T6SSi_tssD, T6SSi_tssK, T6SSi_tssH, T6SSi_tssL, T6SSi_tssE, T6SSi_tssB, T6SSi_tssF, T6SSi_tssI, T6SSi_tssA		15	0
T4aP_15	T4aP	Pa-ELI-Chromosome	Chromosome	2529641	4021431	Multi_loci	0.588	7.26	T4aP_pilQ, T4aP_pilT, T4aP_pilA, T4aP_pilC, T2SS_gspO, T4aP_pilB	T4aP_pilW, T4aP_pilM, T4aP_pilN, T4aP_fimT	6	4
T5aSS_1	T5aSS	Pa-ELI-P1	Plasmid	149150	151123	Single_locus	1	1	T5aSS_PF03797		1	0
T5aSS_2	T5aSS	Pa-ELI-P1	Plasmid	349424	352264	Single_locus	1	1	T5aSS_PF03797		1	0
T4SS_typeF_4	T4SS_typeF	Pa-ELI-P2	Plasmid	157837	184231	Single_locus	0.923	7.1	T4SS_t4cp2, T4SS_virb4, T4SS_MOBF	T4SS_F_traW, T4SS_F_traH, T4SS_F_traF, T4SS_F_traN, T4SS_F_traV, T4SS_F_traK, T4SS_F_traU, T4SS_F_traL, T4SS_F_traE	3	9
T5aSS_3	T5aSS	Pa-ELI-P3	Plasmid	90	2723	Single_locus	1	1	T5aSS_PF03797		1	0

T1SS_1	T1SS	Pa-ELI-P3	Plasmid	3144	8074	Single_locus	1	3	T1SS_abc, T1SS_mfp, T1SS_omf	3	0
T1SS_2	T1SS	Pa-ELI-P3	Plasmid	30012	34780	Single_locus	1	3	T1SS_abc, T1SS_mfp, T1SS_omf	3	0

Table S3: Predicted secondary metabolite regions for *P. agglomerans* ELIV (AntiSMASH).

AntiSMASH Region ID	Replicon name	Replicon type	Begin	End	Length	State	Region type	MIBiG	Completion	Product	Type
1	Pa-ELI-Chromosome.1	Chromosome	554084	580340	26257	Complete	Thiopeptide	BGC0000781	0.142857	O-antigen	Saccharide
2	Pa-ELI-Chromosome.1	Chromosome	646276	706127	59852	Complete	Hserlactone, arylpolyene	BGC0002008	0.944444	Aryl polyenes	Other
3	Pa-ELI-Chromosome.1	Chromosome	1165388	1187554	22167	Complete	Redox-cofactor	BGC0001100	0.133333	Lankacidin C	NRP+Polyketide
4	Pa-ELI-Chromosome.1	Chromosome	3718661	3772351	53691	Complete	NRP-metallophore, NRPS	BGC0002476	1	Enterobactin	NRP
5	Pa-ELI-Chromosome.1	Chromosome	3849647	3870285	20639	Complete	Hserlactone				
1	Pa-ELI-P1.1	Plasmid	364216	387777	23562	Complete	Terpene	BGC0000656	1	Zeaxanthin	Terpene
2	Pa-ELI-P1.1	Plasmid	560051	589877	29827	Complete	NI-siderophore	BGC0001572	1*	Desferrioxamine E	Other
1	Pa-ELI-P2.1	Plasmid	194151	289050	94900	Complete	NRP-metallophore, NRPS	BGC0002475	0.5	Enantio-pyochelin	NRP
